# Estimation of Psychological Impairment and Coping Strategies during COVID-19 Pandemic among University Students in Saudi Arabia: A Large Regional Analysis

**DOI:** 10.3390/ijerph192114282

**Published:** 2022-11-01

**Authors:** Tauqeer Hussain Mallhi, Naveed Ahmad, Muhammad Salman, Nida Tanveer, Shahid Shah, Muhammad Hammad Butt, Ahmed D. Alatawi, Nasser Hadal Alotaibi, Hidayat Ur Rahman, Abdulaziz Ibrahim Alzarea, Abdullah Salah Alanazi, Mohammad Saeed Alzahrani, Sameer Alshehri, Ahmed Aljabri, Yusra Habib Khan

**Affiliations:** 1 Department of Clinical Pharmacy, College of Pharmacy, Jouf University, Sakaka 72341, Saudi Arabia; 2 Department of Pharmaceutics, College of Pharmacy, Jouf University, Sakaka 72341, Saudi Arabia; 3 Institute of Pharmacy, Faculty of Pharmaceutical and Allied Health Sciences, Lahore College for Women University, Lahore 54000, Pakistan; 4 Institute of Molecular Cardiology, University of Louisville, Louisville, KY 40202, USA; 5 Department of Pharmacy Practice, Faculty of Pharmaceutical Sciences, Government College University Faisalabad, Faisalabad 38000, Pakistan; 6 Department of Medicinal Chemistry, Faculty of Pharmacy, Uppsala University, 75123 Uppsala, Sweden; 7 Department of Clinical Pharmacy, College of Pharmacy, Taif University, P.O. Box 11099, Taif 21944, Saudi Arabia; 8 Department of Pharmaceutics and Industrial Pharmacy, College of Pharmacy, Taif University, P.O. Box 11099, Taif 21944, Saudi Arabia; 9 Department of Pharmacy Practice, Faculty of Pharmacy, King Abdulaziz University, Jeddah 22254, Saudi Arabia

**Keywords:** psychological health, students, universities, depression, anxiety, stress, coping strategies, mental health, psychological

## Abstract

Background: The COVID-19 pandemic and associated restrictive measures have substantially affected educational processes around the globe, resulting in psychological distress among students. The mental health of students in higher education is of paramount importance, and the COVID-19 pandemic has brought this vulnerable population into renewed focus. In this context, the evaluation of students‘ mental health at educational institutes has gained invaluable popularity during the COVID-19 pandemic. This study aimed to ascertain the psychological health and coping strategies among students from a higher education institute in Saudi Arabia. Methods: An online study instrument was used to assess anxiety (Generalized Anxiety Disorder-7, GAD-7), depression (Patient Health Questionnaire-9, PHQ-9), post-traumatic stress disorder—PTSD (Impact of Event Scale-Revised, IES-R) and coping strategies (Brief-COPE). The severity of the psychological distress was classified as per the scoring criteria and correlated with demographics using appropriate statistical methods. Results: Of 1074 students (age 21.1 ± 2.1 years), 12.9% and 9.7% had severe anxiety and depression, respectively. The mean anxiety and depression scores were 7.50 ± 5.51 and 9.31 ± 6.72, respectively. About one-third (32%) of students reported suicidal ideation, with 8.4% students having such thoughts nearly every day. The average PTSD score was 21.64 ± 17.63, where avoidance scored higher (8.10 ± 6.94) than intrusion and hyperarousal. There was no association of anxiety, depression and PTSD score with the demographics of the study participants. Religious/spiritual coping (5.43 ± 2.15) was the most adoptive coping mechanism, followed by acceptance (5.15 ± 2.10). Male students were significantly (*p* < 0.05) associated with active copings, instrumental support, planning, humor, acceptance and religious coping. Substance use was the least adopted coping strategy but practiced by a considerable number of students. Conclusions: The long-lasting pandemic situation, onerous protective measures and uncertainties in educational procedures have resulted in a high prevalence of psychological ailments among university students, as indicated in this study. These findings accentuate the urgent need for telepsychiatry and appropriate population-specific mental health services to assess the extent of psychological impairment and to leverage positive coping behaviors among students.

## 1. Introduction

The coronavirus disease 2019 (COVID-19) caused by severe acute respiratory syndrome coronavirus 2 (SARS-CoV-2) is the largest catastrophic calamity of the century. The unpredicted upsurge of COVID-19 has caused unrest resulting in partial or complete lockdowns around the world [1,2]. This disease has posed massive changes in individual’s mental status and psychology in all age groups [3]. The pandemic situation is further worsened by inadequate information about the cause, spread, prevention and treatment. These misleading narratives have resulted in ambiguity and insecurity among the general population, leading to several psychological ailments [4,5]. The psychological problems initiated with fear, depression and anxiety may worsen hysteria and lifelong stigma [6]. Various infectious diseases, including Human Immunodeficiency Virus/Acquired Immunodeficiency Syndrome) HIV/AIDS, Severe Acute Respiratory Syndrome (SARS), Influenza A virus subtype H1N1 and Ebola, have been found to be associated with anxiety, depression, post-traumatic stress and other psychological problems in both infected and noninfected populations [7,8].

The COVID-19 pandemic has substantially impacted the education sector amid imposed restrictions to curb the spread of COVID-19. Changes in the mode of teaching, instruction and evaluation harm the psychological health of the students, as well as the instructors [9].

The first case of COVID-19 was reported on 2 March 2020 in the Kingdom of Saudi Arabia (KSA) [10]. The condition worsened rapidly, and a partial lockdown was imposed throughout the country. Educational institutions switched to e-learning, and examinations were also conducted online, as per the instructions of the Ministry of Education, Saudi Arabia [10]. However, the lockdown was gradually lifted, and offices were opened with precautions on 21 June 2020 [11]. At the beginning of the academic year 2020–2021, universities implemented new Ministry of Education guidelines to contain COVID-19. According to these guidelines, lectures were delivered online while practical lab work and examinations were conducted on-campus with strict precautionary measures [12]. Over the past year, the students in Saudi universities have faced unprecedented circumstances due to the online mode of education and to hybrid/mixed-mode education, as well as uncertainties in the evaluation and enrollment procedures. In addition, the learning process is compromised due to a lack of face-to-face interactions with instructors; technical problems during online lectures and limited group learning, cocurricular and social activities.

Previous studies investigated the impact of COVID-19 on psychological health among university students in different countries [12,13,14,15,16,17,18]. Most of these studies were conducted during the initial months of the pandemic when strict restrictions were in action and it was found that moderate to extremely severe psychological distress was present among university students [16]. However, there is a dearth of investigations ascertaining the psychological impact, particularly post-trauma mental health and adopted coping strategies among university students in KSA, especially at the time when the lockdown measures were maximumly relaxed. Such studies will help the authorities to ascertain the mental health of students in the presence or absence of government mandates during the pandemic. In this context, the current study was aimed to investigate the generalized anxiety disorder (GAD), depression, post-traumatic stress disorder (PTSD) and coping strategies among university students. The findings of the current study will underscore the crucial aspects that will help the Ministry of Education and Health to redesign and implement focused policies during the current or future outbreaks for this vulnerable population from higher education institutes.

## 2. Materials and Methods

### 2.1. Study Design, Settings and Subjects

**Figure 1:**
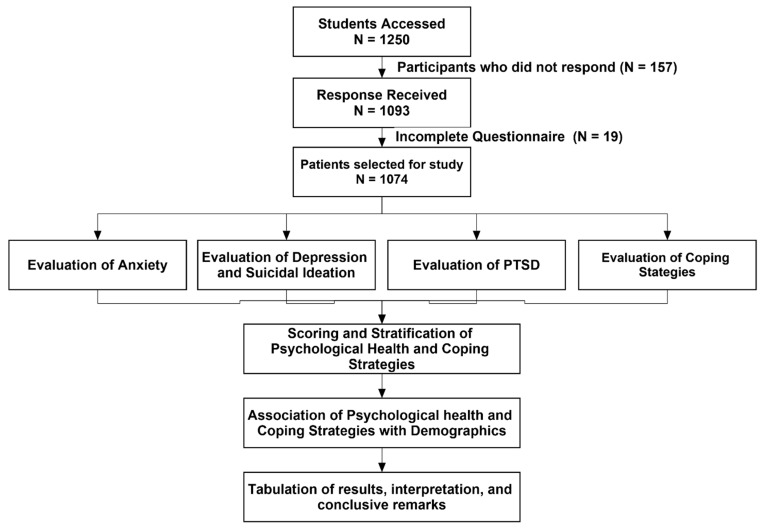
Study flow diagram.

A cross-sectional, descriptive, web-based study was conducted during the second semester of academic year 2021 among students at Jouf University, Kingdom of Saudi Arabia (KSA). The university was following a mixed mode of education at the time of the data collection. Jouf University is largest educational institute in the Al-Jouf region and has a diverse population of the students across the country. The study was conducted on a large scale to achieve a good sample size. All the university students, regardless of educational program, age and gender, were included in this study. However, we excluded those who were not university students, had already graduated and those unwilling to participate in the study. The flow diagram for the current study is described in Figure 1.

### 2.2. Ethical Approval and Considerations

The current study protocol was approved by the Local Committee of Bioethics (LCBE), Deanship of Research at Jouf University, KSA (Reference no. 03-01-43). Moreover, the study was conducted according to the guidelines of the Declaration of Helsinki, and an online consent was obtained from the study participants.

### 2.3. Study Instruments

The study tool consisted of various questions on sociodemographic details (age, gender, semester, study year, city, college and educational status). Moreover, anxiety, depression, post-traumatic stress disorder and coping strategies adopted by the students were assessed by the Generalized Anxiety Disorder Scale (GAD-7), Patient Health Questionnaire (PHQ-9), Impact of Events Scale-Revised (IES-R) and Brief-COPE, respectively. The suicidal ideation rate was assessed using the ninth item of the PHQ-9 Scale. The study instruments were administered to participants in a validated Arabic language [19,20,21].

### 2.4. Outcome Measures

The GAD-7 Scale was used to assess the anxiety among students. This scale consists of seven questions total, and each item ranges from a score of zero to three, yielding a minimum of zero and maximum of twenty-one. Each question consists of four options: the “not at all” option is represented by a score of zero, several days (less than a week) is represented by a score of one, over half the days (more than a week) is represented by a score of two and nearly every day is represented by a score of three. Scores < 5, 5–9, 10–14 and ≥15 were considered the cutoff points for less, mild, moderate and severe anxiety, respectively [4].

The PHQ-9 Scale was used to ascertain the extent of depression. The PHQ scale consists of nine questions, each of which was scored between zero to three and represents the same criteria as was used in GAD-7 scoring, yielding a minimum of zero and maximum of twenty-seven score. Scores of ≤4, 5–9, 10–14, 15–19 and ≥20 reflect minimal, mild, moderate, moderately severe and severe depression, respectively [4]. The ninth item of the PHQ-9 Scale indicates the presence of suicidal ideation. This item phrases the question as “Over the past two weeks, how often have you been bothered by thoughts that you would be better off dead or of hurting yourself in some way?”. The participants responded on a scale of “not at all”, “several days”, “more than half the days” and “nearly every day”. This item is often used in research studies to evaluate suicidal behavior. Previous studies have also reported that high levels of suicidal ideation, indicated by the ninth item of PHQ-9, is a robust predictor of suicide attempts and deaths [22,23,24,25].

The IES-R consists of 22 items and was used to assess PTSD among the study participants. The items are rated on a 5-point scale ranging from 0 (“not at all”) to 4 (“extremely”). The IES-R yields a total score of 88 (ranging from 0 to 88), and subscale scores were also calculated for the intrusion (items 1, 2, 3, 6, 9, 14, 16 and 20); avoidance (items 5, 7, 8, 11, 12, 13, 17 and 22) and hyperarousal (items 4, 10, 15, 18, 19 and 21) subscales.

The Brief-COPE questionnaire is most commonly used instrument to assess the adopted coping strategies. It is a validated twenty-eight-item questionnaire that evaluates numerous ways to cope with a stressful life event, such as the current COVID-19 pandemic. Each item has a score of one to four, yielding a minimum score of twenty-eight and maximum of one hundred and twelve. Higher scores indicated a higher tendency to implement the corresponding coping style. The Brief-Cope questionnaire consists of fourteen different coping strategies or facets: self-distraction, active coping, denial, substance use, use of emotional support, use of instrumental support, behavioral disengagement, venting, positive reframing, planning, humor, acceptance, religion and self-blame.

### 2.5. Validation of Study Instrument

The contents of the study instruments were reviewed by a panel of 7 experts from psychology, medicine, pharmacy, public health and health policy disciplines. All the suggested changes from experts were incorporated in the final data collection tool and a subsequent approval was obtained. Forward and backward translational accuracy was also ensured. The accuracy of the translation was assessed by three language experts, and interrater reliability was applied to find a proper translation. Furthermore, the pilot scale study was performed among 50 participants to validate the study instrument. The Cronbach’s alpha value was more than 0.70 for each scale, which represented the internal consistency and validity of the scale. However, the data of all pilot scale participants were excluded from the final analysis. It is pertinent to note that the Arabic versions of the PHQ-9, GAD-7, IES-R and Brief COPE were previously validated in the Saudi population [19,26,27]. We also compared the translated version of each scale with these validated tools.

### 2.6. Data Analysis

All the collected data were screened and analyzed using SPSS version 23. All the descriptive statistics of the categorical variables were computed for frequencies and percentages, while the means and standard deviations (*SD*) were calculated for all the tested scales. The differences in the mean scores of all the dichotomous variables were measured through the *t*-test. For multiple categorical variables (e.g., gender group, age groups, education, marital status, etc.), one-way ANOVA was used, with Tukey’s post hoc for homogeneous variances. The chi-square test was performed to assess differences among the grouped variables. A *p*-value < 0.05 was considered statistically significant throughout the study analysis.

## 3. Results

### 3.1. Characteristics of Study Participants

Of the 1250 students accessed for this study, 1093 students responded to the survey (response rate: 87.4%). However, 1074 participants were included in the final analysis after the exclusion of 19 incomplete responses. The mean age of the respondents was 21.06 ± 2.09 (range 17–29). There was a preponderance of female students (74.3%) from the health sciences (38.7%). Around 18% of the study participants reported to be infected with SARS-CoV-2, and a wide majority (81.8%) of the students reported having a family member, relative, neighbor or acquaintance suffering from COVID-19.

### 3.2. Anxiety and Depression

The mean GAD-7 score was 7.50 ± 5.51. The prevalence of the generalized anxiety (score ≥ 10) was 32.1% among the study participants. Moreover, the prevalence of mild, moderate and severe anxiety was 32%, 19.2% and 12.9%. As shown in Table 1, there was no significant difference (*p* > 0.05) in the anxiety scores among the demographic variables. The mean PHQ-9 score of the study participants was 9.31 ± 6.72. Regarding the severity of depression, 27.7%, 31.5%, 20.6%, 10.6% and 9.7% of the students were found to have minimal/none, mild, moderate, moderately severe and severe depression, respectively. However, the depression score was not associated with the demographics (Table 1).

### 3.3. Suicidal Ideation/Self-Harm

The prevalence of suicidal ideation was 32% in our sample. Regarding the severity of the suicidal thoughts/self-injury, 16.5% reported them occurring several days, 7.2% over half the days and 8.4% had them nearly every day in the past two weeks. In the chi-square analysis, no significant difference (*p* > 0.05) of the suicidal ideation rate was observed among the study demographics.

### 3.4. Post-Traumatic Stress Disorder (PTSD)

**Figure 2:**
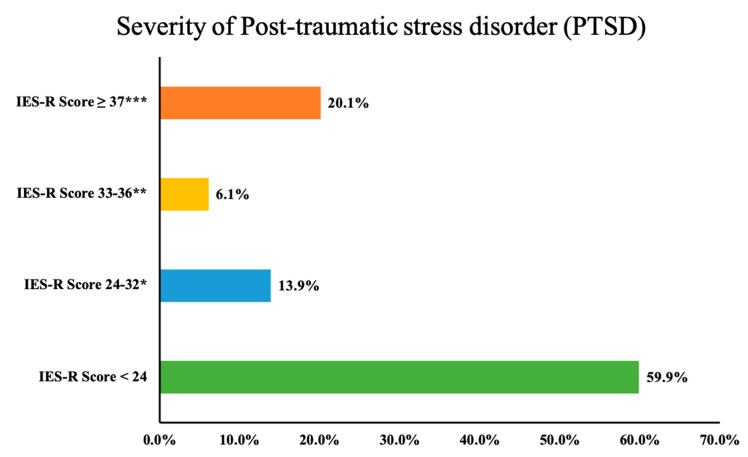
Severity of PTSD among the study participants (* Those with scores this high who do not have full PTSD will have partial PTSD or at least some of the symptoms. ** The best cutoff for a probable diagnosis of PTSD. *** It is high enough to suppress the immune system’s functioning (even 10 years after an impact event).

The mean PTSD score was 21.64 ± 17.63 (subscale scores: Avoidance: 8.10 ± 6.94, Intrusion: 7.49 ± 6.63 and Hyperarousal: 6.05 ± 5.31). Using the best cutoff scores for the probable diagnosis of PTSD (score ≥ 33), we found 26.2% students suffering from PTSD. As depicted in Figure 2, around 20% of the students had an IES-R score of ≥37, a high enough score to suppress the immune system’s functioning. However, the PTSD score was not significantly associated with the demographic features of the study participants (Table 1).

### 3.5. Coping Strategies Adopted by the Respondents

As shown in Table 2, the most common coping method was religious/spiritual coping (5.43 ± 2.15), followed by acceptance (5.15 ± 2.10), active coping (4.65 ± 1.86) and positive reframing (4.47 ± 1.97). Regarding the intra-demographic differences of the coping strategies, male students had significantly higher scores in active copings (*p* = 0.014), instrumental support (*p* = 0.009), planning (*p* = 0.001), humor (*p* = 0.015), acceptance (*p* = 0.004) and religious coping (*p* = 0.044). Among the different education categories, a significant difference was seen for positive reframing, planning, acceptance and religious coping. However, the year of study had no influence (*p* > 0.05) on the coping strategies adopted by the students. In multiple comparisons using Tukey’s HSD (Table 3), only engineering students had significantly higher scores for positive reframing (*p* = 0.022), planning (*p* = 0.005) and religious coping (*p* = 0.016) than the students belonging to the business and humanities education group. Furthermore, students from the business and humanities category had significantly lower religious coping scores than those from the natural sciences education group (*p* = 0.011). There was no significant difference in coping methods between students who got infected with COVID-19 and those who did not, except for the substance use (*p* = 0.043).

## 4. Discussion

Mental and psychological health-related issues are the leading impediment to academic success [28]. University students comprise a population that is considered particularly vulnerable to mental health intricacies [29]. Mental illness can affect students’ motivation, concentration and social interactions—crucial factors for students to succeed in higher education. In this context, quality data on the psychological health of this vulnerable population during the COVID-19 pandemic is the need of the hour. There is a dearth of investigations evaluating the psychological health of university students during the pandemic. Most of the studies conducted in KSA have included university students from specific disciplines such as medicine, pharmacy or nursing [30,31,32,33]. Other studies among the overall university students ascertained only anxiety, depression and stress by using various questionnaires [34,35,36,37,38]. The coping strategies adopted by the students during the ongoing pandemic were assessed by only two studies using self-constructed tools [39,40]. We did not come across any study quantifying the extent of anxiety, depression, post-traumatic mental health and coping strategies in the same population of the students. Moreover, the available data do not represent the psychological illustration of students from the northern region of KSA, the least developed area in the country. In this context, this study is the first of its kind to ascertain the comprehensive psychological health, post-traumatic stress and coping strategies among a diverse group of university students from the Al-Jouf region. Additionally, this study used pre-validated and commonly used data collection tools, which will further strengthen the implications of the findings. Since the students belong to a vulnerable group for various mental health issues [41], this study was aimed to provide insight on the psychological health among these students during the COVID-19 pandemic, which would help health and education authorities in designing and implementing targeted policies during the current or any future catastrophic event.

This study revealed a high prevalence of anxiety, depression and post-traumatic stress disorders among university students. The studies previously conducted in KSA used variable tools for the assessment of anxiety, depression and stress, i.e., DASS-21 (Depression, Anxiety and Stress Scale) [34,35], GAD-7 and PHQ-9 [32,36,37]. However, we compared our findings with studies using similar tools for the assessment of mental health among university students, regardless of their discipline of education.

This study demonstrated a high prevalence of anxiety and depression among university students, where more than one-third had moderate to severe anxiety and depression. These findings are consistent with the other studies conducted in KSA [31,37,38]. The available data indicated the prevalence of severe anxiety among students to range from 19% to 24%, which is comparatively higher than reported in the current study (12.9%). These disparate findings might be attributed to the timing of the data collection, as other studies were conducted during the lockdown, and data collection in our study was initiated after relaxing the strict preventive measures. Moreover, the population in one study was pharmacy students [31], and the association of healthcare students with deteriorated mental health has already been established [20,21]. A recent literature review of 37 studies indicated that around one-third of students had anxiety during the early phases of the pandemic [42]. Another meta-analysis of 36 studies showed a pooled prevalence of 41% among university students, where the highest prevalence was observed among students from the USA (56%), Europe (51%) and Asia (33%) [43]. The prevalence of severe depression in our study (9.7%) corresponds to the results of Alhadi and colleagues (11.2%) [20]. The COVID-19 pandemic has also been observed to be associated with suicidal behavior among university students [44]. Approximately one-third of the study participants experienced suicidal ideation in our study, where 8.4% had such thoughts nearly every day in the past two weeks. These results correspond to the findings of Alhadi and Colleagues, where 6% of Saudi students had suicidal wishes nearly every day [38]. A recent investigation among Pakistani students also demonstrated similar findings, where 32% of university students had thoughts about death and self-harm during the pandemic. These thoughts or intentions were equally distributed among the demographics [45]. It is worth mentioning that a substantial number of students are still experiencing a varying severity of anxiety and depression, even after relaxing the movement restrictive measures. These findings necessitate the need for an urgent response from the university administration and health and educational authorities. The provisional mobile app for mental health service for students by the university administration has been reported in the literature [46].

The relationship of the geographical location of the students and severity of anxiety has already been established, where students from the eastern region (urban) of the KSA had significantly higher anxiety and depression scores [20]. However, the authors did not describe the factors that may be linked to such an association. In contrast, a study from China reported a higher detection rate of anxiety among rural residents than that of the general population in the country [47]. Most of the students at Jouf University reside in rural territories, which may be related to the high detection rate of anxiety and depression in our cohort. The variations of the findings across the literature underscore the need for a regional analysis so a firm conclusion could be drawn. However, a relationship of the geographical location with mental health is also confounded by various factors. The implementation of strict and vigorous measures and the substantial rise of COVID-19 cases in urban, developed and congested regions may contribute to higher levels of psychological disorders among the residents. On the other hand, the lack of facilities, low socioeconomic status and limited access to healthcare facilities may also be associated with psychological distress among rural residents. At the same time, less detection of anxiety among rural residents might be attributed to less prevalent cases of COVID-19 and a wide range of activities in a relatively safe and isolated environment. Taken together, the existing literature represents wide disparities across the available literature that warrant more research considering the geographical aspect of the study population. The existing national and international literature has elaborated a significant association of the female gender, young age, a history of COVID-19-positive cases among family and friends, chronic diseases, previous mental illnesses, living alone, healthcare students and students with learning difficulties with psychological ailments during the pandemic [20,31,32,37,48]. However, the demographic features were not found to be associated with the psychological illness scores in our study.

Post-traumatic stress was observed in a substantial number of cases, where 20% of the population had a PTSD score >37, a high enough score to suppress the functioning of the immune system, even 10 years after an impact event. These results are in line with the findings of another study conducted among Saudi students, where 23.8% of the population indicated severe PTSD scores [35]. A recent meta-analysis of six studies from China, the USA and France pooled the prevalence of PTSD at 23% among university students and suggested its relationship with health and wellbeing, as well as the quality of education [49]. Of the three facets of PTSD, avoidance scored highest, followed by intrusion and hyperarousal. The avoidance cluster of PTSD symptoms is categorized as an attempt to avoid distressing memories, thoughts or feelings, as well as external reminders such as conversations about the traumatic event that bring the event to mind. Our analysis revealed that students are maximally attempting to avoid any trigger that recalls the COVID-19 pandemic. Additionally, a high intrusion score among students indicates their inability to keep memories of the event from returning, and they are experiencing intrusive thoughts or recollections and recurrent dreams or flashbacks of the trauma. A recent study conducted in the Middle East and North African (MENA) regions also demonstrated a high IES-R score in the general population [50]. Since data regarding the impact of the event on the psychological health of the students are scarce, the results of our study not only fill the literature gap but also provide pivotal implications for health and educational authorities. It is pertinent to discuss that one-quarter of the students scored ≥33 on the IES-R Scale, even two years after the outbreak. These results explicitly indicate that the psychological health of students is still deteriorating, which entails immediate measures, including counseling sessions and addressing associated stressors. Psychoeducation or cognitive behavior therapies have been well = recommended during such circumstances [51]. The implementation of community-based strategies, telehealth services and awareness programs for self-relaxation and self-care can provide promising psychological support to this vulnerable population [52].

The available studies have elaborated that students and their families are markedly affected by the lack of support from educational institutes, prolonged closure of schools, limited facilities for online education and uncertainty about the students‘ evaluations and enrollment procedures [53,54]. All the public and private educational institutes were immediately closed following the first report of COVID-19 in the KSA. However, the education process was not halted at the university level, and all the teaching activities were continued virtually through Blackboard (a web-based virtual learning environment and learning management system). The lack of good internet service in remote areas might have resulted in a panicked state among students, fostering anxiety, depression and uncertainty in this population. It might be a possible reason for the high scores of psychological illnesses in our study. These results necessitate the need for proactive support regarding information technology for students, specifically those residing in rural vicinities.

Unfortunately, limited data are available on how students within the KSA are coping with the COVID-19 pandemic and the extent of its ramifications on their mental health and well-being. This study found religious, acceptance, active and positive reframing as the most commonly used coping strategies among university students. These findings are in concordance with other studies conducted among university students in Pakistan, the Middle East and North Africa [4,39]. Interestingly, the demographic features of the students, such as gender, discipline of study and history of COVID-19 among the participants or family and friends, had a significant association with the adopted type of coping strategies. The coping strategies can be stratified into adaptive/approach and maladaptive/avoidant copings, where the latter is not ideal in dealing with psychological impairments and may lead to major depressive symptoms, more severe anxiety, low life satisfaction, negative thinking and poorer physical health. Fortunately, the scores for adaptive or positive copings were higher than maladaptive or avoidant copings in our study. It is important to note that a previous study from the KSA reported mental disengagement as the second-most common coping strategy among university students [40]. Similarly, mental disengagement was also reported as the second-most common adopted coping strategy among university students in the KSA by a multinational study [39]. “Acceptance” was the most commonly used coping strategy in these two studies. Mental disengagement involves directing attention and effort toward the goal of alleviating negative emotions by engaging in substitute activities to keep one’s mind from ongoing stressors and is classified as negative, maladaptive or avoidant coping. There is a high propensity of developing lower well-being or lower life satisfaction among students practicing mental disengagement to mitigate anxiety or stress. Our study also revealed a high score (>4.3) for self-distraction, which was close to positive reframing (>4.5). In addition, the proportion of students using behavioral disengagement and substance use as coping mechanisms should not be disregarded in the current study. Recent investigations have also confirmed that PTSD symptoms are significantly linked with substance use regardless of gender [55]. Maladaptive coping behaviors are significant predictors of depression among young adults. Our study participants seem to dispel stress temporarily through negative coping mechanisms but may have major depressive and anxiety symptoms at a later stage. The identification of students‘ coping behavior is of paramount importance to inform the planning and design of support systems. A prime responsibility lies on the shoulders of educational institutes to develop culturally sensitive mental health services that could leverage natural positive coping behaviors among students. These participatory models have been used previously by educational institutes to address the mental health of their students [56,57].

It is incumbent to note that the curriculum and learning outcomes of an educational program are linked with the teaching and evaluation methods. The COVID-19 pandemic has interfered with the pedagogy, as well as the assessment, of students [58,59]. In this context, there is a need to consider modifications or revisions in the education process during a natural crisis. There should be a radical transformation at various educational levels, i.e., from curriculum to pedagogy, from instructor to students and from learning to assessment. Courses that are suitable for online learning should be developed during such times when students are confined to their homes. Modifications of the curriculum and learning outcomes according to the virtual classrooms will also aid in improving the mental health of students during disease outbreaks.

### 4.1. Study Limitations

A few shortcomings in this study should be considered while interpreting the results. The use of a self-reported questionnaire may be associated with respondents‘ bias, introspective ability or misreporting of data. The cross-sectional study design also limits psychological responses at a specific time. Since data were collected at the time during which the lockdown measures were significantly relaxed, there is a possibility of different results if the data were collected during the acute phase of the pandemic when students were quarantined at their homes. The snowballing sampling strategy is another limitation, and there is a possibility of limited participation of students who are not active on social network applications. The study population was from one higher education institution, and the findings may not be generalizable with other regions of the country. Nevertheless, given the nationwide similarities in universities transitioning to virtual classes and similar precautionary orders, we expect a reasonable generalizability of these results. The clinical diagnoses of anxiety, depression and PTSD were not made according to the Diagnostic and Statistical Manual of Mental Disorders (DSM-V). Furthermore, participants who were found to have severe anxiety, depression, PTSD or suicidal ideation/self-harm could not be referred to a consultant psychiatrist for a detailed evaluation, due to the anonymized self-completed questionnaire. Despite this, the large analysis provided valuable insights about psychological health and the adopted copings among students from the higher education sector.

### 4.2. Implications for Future Research and Practice

In light of our findings, there is a need for immediate attention and support for students with mental health issues. It is also essential to assess psychological impairments and psychosocial plans among students through large-scale studies so effective support mechanisms can be implemented during the recovery phase, as well as for similar events in the future. The findings of this study, along with the results of other similar investigations, necessitate the importance of telepsychiatry in such catastrophic events. Since psychological impairments may linger even after the end of the pandemic, future studies should also focus on the mental health of students at later stages of the COVID-19 pandemic.

Academic institutions need to screen their students periodically to identify the prevalence of psychological disorders and should consider coordinated approaches to address them. This can be achieved by establishing “student psychological and counselling services” to ensure the provision of appropriate psychological care. Educational institutes should consider flexible and effective approaches for virtual education without compromising the competencies of the learning. Contingency planning for the educational process based on the feedback of stakeholders will not only aid in the continuation of education but will also provide directions to students during natural disaster events. Future research should focus on the longitudinal follow-up of students with mental disorders, covariates associated with psychological health and their relationship with the learning experience.

## 5. Conclusions

Our findings indicated the high prevalence of psychological disorders among university students, where one-third of students had generalized anxiety, moderate-to-severe depression and suicidal ideation. More than one-fourth of the study population scored ≥33 on the IES-R Scale, indicating a profound proportion of students with PTSD. Anxiety, depression and PTSD were not associated with the demographics of the study participants. Religious/spiritual and acceptance copings were the highest adaptive mechanisms to mitigate psychological issues, while substance use was the least adopted but practiced in a considerable number of students. These findings necessitate the need for telepsychiatry and appropriate population-specific mental health services to assess the extent of the psychological impairments and to leverage positive coping behaviors among students. Tailored coping strategies would be of great value to address the needs of students and improve their psychological resilience. Future research should focus on the chronic impact of the pandemic on mental health, barriers to virtual classrooms, including revisions of the curricula and learning outcomes, precipitating factors of poor mental health and participatory models to combat the psychological issues during the current, as well as future, health disastrous events.

## Figures and Tables

**Table 1:** Psychological assessment among the study participants and its association with the demographic features.

Demographics	Subgroups	N (%)	Anxiety Score	Depression Score	PTSD Score
**Overall Score**			7.50 ± 5.51	9.31 ± 6.72	21.64 ± 17.63
**Age**	≤20 years	449 (41.8)	7.70 ± 5.69	9.14 ± 6.84	22.04 ± 18.48
	21–25 years	603 (56.1)	7.37 ± 5.39	9.48 ± 6.66	21.51 ± 17.10
	≥26 years	22 (2.0)	6.73 ± 5.18	8.41 ± 5.76	17.27 ± 13.88
	*p*-value	--	0.512	0.587	0.312 *
**Gender**	Male	276 (25.7)	7.51 ± 5.66	9.56 ± 6.64	22.01 ± 17.58
	Female	798 (74.3)	7.49 ± 5.46	9.23 ± 6.75	21.52 ± 17.66
	*p*-value	--	0.959	0.484	0.69
**Education**	Health Sciences	416 (38.7)	7.30 ± 5.61	9.15 ± 6.82	21.55 ± 17.29
	Engineering	195 (18.2)	7.60 ± 5.94	9.48 ± 7.61	24.43 ± 20.20
	Natural Sciences	168 (15.6)295 (27.5)	7.83 ± 5.25	9.29 ± 6.19	21.72 ± 17.09
	Business and Humanities		7.50 ± 5.29	9.44 ± 6.23	19.89 ± 16.42
	*p*-value	--	0.760 *	0.930 *	0.075 *
**Year of study**	First	269 (25.0)	7.57 ± 5.77	8.87 ± 6.92	22.24 ± 19.28
	Second	257 (23.9)	7.62 ± 5.39	9.45 ± 6.77	22.47 ± 17.98
	Third	248 (23.1)	7.62 ± 5.44	9.54 ± 6.42	21.67 ± 17.32
	Forth	300 (27.9)	7.22 ± 5.45	9.41 ± 6.74	20.37 ± 15.99
	*p*-value		0.792	0.656	0.451 *
**Family member, relative or acquaintances got COVID-19**	Yes	878 (81.8)	8.08 ± 6.05	9.76 ± 7.48	24.02 ± 19.51
	No	196 (18.2)	7.37 ± 5.38	9.21 ± 6.53	21.11 ± 17.15
	*p*-value	--	0.1	0.346	0.055
**Infected with COVID-19?**	Yes	189 (17.6)	7.64 ± 5.49	9.40 ± 6.71	21.83 ± 17.50
	No	885 (82.4)	6.84 ± 5.58	8.90 ± 6.75	20.76 ± 18.28
	*p*-value		0.07	0.35	0.45

* Welch’s ANOVA was used instead of the classic ANOVA, as the assumption of homogeneity of the variances was violated.

**Table 2:** Coping strategies adopted by the study participants.

Variable	Subgroups	Self-Distraction	Active Coping	Denial	Substance Use	Emotional Support	Instrumental Support	Behavioral Disengagement	Venting	Positive Reframing	Planning	Humor	Acceptance	Religion	Self-Blame
**Overall**	--	4.30 ± 1.742	4.65 ± 1.86	3.40 ± 1.71	2.64 ± 1.32	4.02 ± 1.80	4.01 ± 1.94	3.32 ± 1.59	38.3 ± 1.71	4.47 ± 1.97	4.51 ± 1.96	36.8 ± 1.81	5.15 ± 2.10	5.43 ± 2.15	3.59 ± 1.82
**Age**	≤20 years	4.29 ± 1.79	4.70 ± 1.85	3.40 ± 1.76	2.60 ± 1.27	4.03 ± 1.81	3.94 ± 1.91	3.31 ± 1.64	3.82 ± 1.72	4.57 ± 2.03	4.57 ± 2.02	3.65 ± 1.80	5.13 ± 2.10	5.49 ± 2.19	3.58 ± 1.81
	21–25 years	4.31 ± 1.72	4.59 ± 1.86	3.40 ± 1.68	2.66 ± 1.36	3.99 ± 1.79	4.04 ± 1.94	3.34 ± 1.56	3.83 ± 1.70	4.41 ± 1.92	4.46 ± 1.92	3.71 ± 1.82	5.16 ± 2.09	5.39 ± 2.13	3.62 ± 1.82
	≥ 26 years	4.14 ± 1.49	5.00 ± 2.02	3.45 ± 1.71	2.64 ± 1.00	4.68 ± 1.94	4.59 ± 2.20	3.05 ± 1.36	4.00 ± 1.75	4.14 ± 1.98	4.68 ± 1.86	3.23 ± 1.60	5.45 ± 2.30	5.64 ± 2.30	3.14 ± 1.86
	*p*-value	0.882	0.426	0.989	0.721	0.209	0.251	0.681	0.887	0.299	0.602	0.42	0.769	0.696	0.458
**Gender**	Male	4.35 ± 1.77	4.88 ± 1.89	3.41 ± 1.78	2.72 ± 1.44	4.16 ± 1.92	4.28 ± 2.05	3.38 ± 1.72	3.80 ± 1.72	4.59 ± 1.97	4.83 ± 1.90	3.91 ± 1.91	5.46 ± 2.10	5.66 ± 2.10	3.67 ± 1.84
	Female	4.28 ± 1.73	4.56 ± 1.84	3.40 ± 1.69	2.60 ± 1.27	3.97 ± 1.76	3.91 ± 1.89	3.30 ± 1.57	3.84 ± 1.70	4.43 ± 1.97	4.40 ± 1.97	3.60 ± 1.76	5.04 ± 2.09	5.36 ± 2.17	3.57 ± 1.81
	*p*-value	0.574	**0.014**	0.903	0.217	0.129	**0.009**	0.501	0.792	0.232	**0.001**	**0.015**	**0.004**	**0.044**	0.402
**Education**	Health Sciences	4.25 ± 1.79	4.70 ± 1.96	3.37 ± 1.75	2.67 ± 1.39	4.07 ± 1.86	4.10 ± 1.96	3.33 ± 1.61	3.79 ± 1.69	4.48 ± 2.00	4.56 ± 1.94	3.64 ± 1.82	5.14 ± 2.12	5.45 ± 2.12	3.59 ± 1.79
	Engineering	4.44 ± 1.87	4.75 ± 1.96	3.48 ± 1.84	2.71 ± 1.44	4.10 ± 1.82	4.08 ± 2.06	3.38 ± 1.79	3.93 ± 1.90	4.74 ± 2.04	4.81 ± 2.10	3.66 ± 1.93	5.28 ± 2.15	5.70 ± 2.23	3.75 ± 1.92
	Natural Sciences	4.29 ± 1.71	4.55 ± 1.73	3.34 ± 1.67	2.57 ± 1.22	4.07 ± 1.74	4.12 ± 1.91	3.29 ± 1.53	4.01 ± 1.73	4.57 ± 1.99	4.54 ± 2.01	3.83 ± 1.80	5.51 ± 2.11	5.63 ± 2.17	3.63 ± 1.87
	Business and Humanities	4.28 ± 1.60	4.56 ± 1.72	3.43 ± 1.59	2.57 ± 1.18	3.87 ± 1.73	3.78 ± 1.82	3.29 ± 1.46	3.71 ± 1.58	4.22 ± 1.85	4.21 ± 1.84	3.65 ± 1.71	4.88 ± 2.00	5.12 ± 2.11	3.47 ± 1.74
	*p*-value	0.695	0.551	0.849	0.551	0.411	0.095	0.922	0.256	**0.032**	**0.008**	0.681	**0.015**	**0.012**	0.43
**Year of study**	First	4.18 ± 1.74	4.64 ± 1.91	3.23 ± 1.61	2.59 ± 1.30	3.86 ± 1.68	3.87 ± 1.78	3.17 ± 1.57	3.74 ± 1.68	4.53 ± 1.99	4.47 ± 1.97	3.58 ± 1.74	5.04 ± 2.06	5.49 ± 2.22	3.45 ± 1.74
	Second	4.28 ± 1.71	4.67 ± 1.77	3.58 ± 1.78	2.76 ± 1.41	4.05 ± 1.90	4.01 ± 2.03	3.37 ± 1.68	3.95 ± 1.80	4.35 ± 2.00	4.53 ± 2.04	3.70 ± 1.94	5.02 ± 2.18	5.28 ± 2.15	3.66 ± 1.88
	Third	4.37 ± 1.72	4.75 ± 1.86	3.52 ± 1.82	2.70 ± 1.34	4.25 ± 1.87	4.15 ± 2.00	3.44 ± 1.57	3.74 ± 1.59	4.60 ± 1.95	4.59 ± 1.94	3.70 ± 1.76	5.33 ± 2.04	5.65 ± 2.08	3.60 ± 1.78
	Forth	4.36 ± 1.80	4.54 ± 1.86	3.31 ± 1.64	2.51 ± 1.26	3.96 ± 1.75	4.02 ± 1.93	3.32 ± 1.55	3.88 ± 1.74	4.41 ± 1.94	4.45 ± 1.90	3.73 ± 1.79	5.22 ± 2.11	5.34 ± 2.15	3.67 ± 1.86
	*p*-value	0.571	0.603	0.057	0.130	0.094	0.402	0.271	0.423	0.469	0.848	0.765	0.27	0.225	0.446
**Family member, relative or acquaintances got COVID-19**	No	4.37 ± 1.80	4.76 ± 1.95	3.35 ± 1.80	2.80 ± 1.47	4.23 ± 1.91	4.18 ± 1.96	3.39 ± 1.73	3.91 ± 1.84	4.60 ± 1.96	4.78 ± 2.04	3.67 ± 1.78	5.26 ± 2.18	5.55 ± 2.20	3.68 ± 1.91
	Yes	4.28 ± 1.73	4.62 ± 1.84	3.41 ± 1.70	2.60 ± 1.28	3.97 ± 1.77	3.97 ± 1.77	3.31 ± 1.56	3.81 ± 1.68	4.44 ± 1.97	4.45 ± 1.94	3.68 ± 1.82	5.13 ± 2.08	5.41 ± 2.15	3.58 ± 1.79
	*p*-value	0.503	0.364	0.65	0.073	0.081	0.163	0.486	0.438	0.326	**0.031**	0.997	0.441	0.421	0.449
**Infected with COVID-19?**	No	4.32 ± 1.74	4.67 ± 1.86	3.41 ± 1.72	2.67 ± 1.35	4.04 ± 1.81	4.01 ± 1.93	3.35 ± 1.61	3.85 ± 1.71	4.48 ± 1.94	4.53 ± 1.96	3.72 ± 1.83	5.17 ± 2.08	5.45 ± 2.14	3.64 ± 1.83
	Yes	4.20 ± 1.77	4.52 ± 1.84	3.35 ± 1.67	2.48 ± 1.14	3.92 ± 1.75	4.00 ± 1.97	3.16 ± 1.50	3.72 ± 1.69	4.43 ± 2.12	4.42 ± 1.95	3.50 ± 1.71	5.05 ± 2.17	5.37 ± 2.25	3.38 ± 1.71
	*p*-value	0.379	0.319	0.673	**0.043**	0.397	0.942	0.134	0.362	0.753	0.489	0.133	0.456	0.655	0.074

Bold values represent significant *p*-values.

**Table 3:** Multiple comparisons of the coping strategies according to the Discipline of Education.

Variable	Education	Mean Difference	Standard Error	*p*-Value	95% Confidence Interval	
					Lower Bound	Upper Bound
Positive reframing	Health Sciences vs. Engineering	−0.260	0.170	0.420	−0.70	0.18
	Health Sciences vs. Natural Sciences	−0.082	0.179	0.968	−0.54	0.38
	Health Sciences vs. Business and Humanities	0.259	0.149	0.305	−0.12	0.64
	Engineering vs. Natural Sciences	0.178	0.207	0.824	−0.35	0.71
	Engineering vs. Business and Humanities	0.520	0.181	**0.022**	0.05	0.99
	Natural Sciences vs. Business and Humanities	0.342	0.190	0.273	−0.15	0.83
Planning	Health Sciences vs. Engineering	−0.248	0.169	0.460	−0.68	0.19
	Health Sciences vs. Natural Sciences	0.027	0.178	0.999	−0.43	0.49
	Health Sciences vs. Business and Humanities	0.349	0.149	0.088	−0.03	0.73
	Engineering vs. Natural Sciences	0.275	0.205	0.540	−0.25	0.80
	Engineering vs. Business and Humanities	0.597	0.180	**0.005**	0.13	1.06
	Natural Sciences vs. Business and Humanities	0.322	0.189	0.320	−0.16	0.81
Acceptance	Health Sciences vs. Engineering	−0.145	0.181	0.854	−0.61	0.32
	Health Sciences vs. Natural Sciences	−0.369	0.191	0.215	−0.86	0.12
	Health Sciences vs. Business and Humanities	0.256	0.159	0.375	−0.15	0.66
	Engineering vs. Natural Sciences	−0.224	0.220	0.739	−0.79	0.34
	Engineering vs. Business and Humanities	0.401	0.193	0.161	−0.10	0.90
	Natural Sciences vs. Business and Humanities	0.625	0.202	**0.011**	0.11	1.14
Religious coping	Health Sciences vs. Engineering	−0.248	0.186	0.542	−0.73	0.23
	Health Sciences vs. Natural Sciences	−0.177	0.196	0.805	−0.68	0.33
	Health Sciences vs. Business and Humanities	0.339	0.163	0.162	−0.08	0.76
	Engineering vs. Natural Sciences	0.072	0.226	0.989	−0.51	0.65
	Engineering vs. Business and Humanities	0.587	0.198	**0.016**	0.08	1.10
	Natural Sciences vs. Business and Humanities	0.516	0.207	0.063	−0.02	1.05

Bold values represent significant *p*-values.

## Data Availability

Not applicable.
